# Emergent Transcatheter Arterial Embolization via a Transpopliteal Approach for Internal Iliac Artery Injury during Lumbar Disk Surgery in the Prone Position

**DOI:** 10.3400/avd.cr.20-00074

**Published:** 2020-12-25

**Authors:** Kiyoshi Chiba, Yukihisa Ogawa, Kenji Murakami, Shota Kita, Hirotoshi Suzuki, Masahide Komagamine, Kan Nawata, Masahide Chikada, Hiroshi Nishimaki, Takeshi Miyairi

**Affiliations:** 1 Department of Cardiovascular Surgery, St. Marianna University School of Medicine, Kawasaki, Kanagawa, Japan; 2 Department of Radiology, St. Marianna University School of Medicine, Kawasaki, Kanagawa, Japan; 3 Diagnostic Radiology, Hakodate Goryokaku Hospital, Hakodate, Hokkaido, Japan

**Keywords:** lumbar disk surgery, prone position, iatrogenic vascular injury

## Abstract

This report describes a successful case of transcatheter arterial embolization for a critical vascular injury during lumbar disk surgery that resulted in a large retroperitoneal hematoma in a 72-year-old woman. A 4-Fr long sheath was inserted via the right popliteal artery in the prone position. Pelvic angiography revealed a pseudoaneurysm in the right internal iliac artery, which was managed with coil embolization. The patient underwent laparotomy because of abdominal compartment syndrome and was discharged in good condition after rehabilitation. The transpopliteal endovascular approach in the prone position may thus provide the best chance to treat this rare but critical condition.

## Introduction

Lumbar disk surgery is a common operation, and vascular complications are rare. When an iatrogenic vascular injury occurs during lumbar disk surgery in the prone position, it can lead to hemorrhagic shock or abdominal compartment syndrome, and the prone position makes it difficult to achieve hemostasis surgically. This report describes a patient in whom critical vascular injury occurred during lumbar disk surgery, which was successfully treated with transcatheter arterial embolization in the prone position.

## Case Report

A 72-year-old woman developed lumbar pain and right lower extremity numbness due to disk herniation at the L4/5 level. She subsequently underwent lumbar discectomy at the L4/5 intervertebral spaces in the prone position.

After disc removal, she suddenly developed hypotension (60/34 mmHg) and tachycardia (130 bpm) caused by continuous bleeding from the right side of the L4 area, which required emergency endovascular treatment (EVT).

The EVT was performed in the operating room using a mobile C-arm (OEC 9900 Elite; GE Healthcare, Milwaukee, WI, USA), with the patient in the prone position. While the intervertebral yellow ligament can cause secondary spinal cord injury if the patient is turned over, the patient was maintained in the prone position for the EVT because her spinal cord was partially exposed after removing the vertebrae. The right popliteal artery above the patella was punctured using a 20-G elaster needle (Medikit, Tokyo, Japan) under ultrasound, and a 0.035-inch guidewire (Radifocus; Terumo Clinical Supply, Gifu, Japan) was inserted through the needle. A 4-Fr long sheath was then inserted over the guidewire.

**Figure 1:**
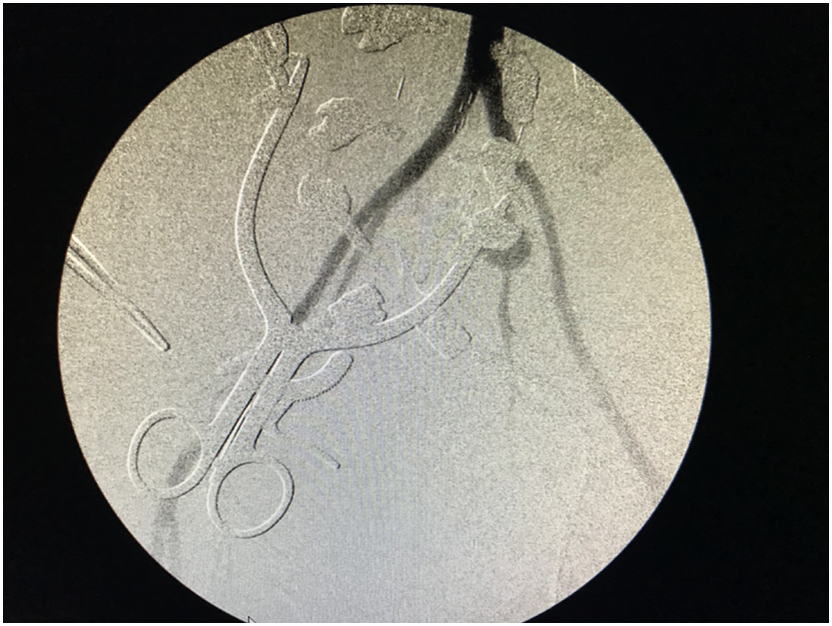
Fig. 1 Pelvic angiography revealed a pseudoaneurysm with extravasation in the right internal iliac artery.

Pelvic angiography revealed a pseudoaneurysm measuring approximately 40 mm with a healthy landing zone of more than 10 mm in the right internal iliac artery (**Fig. 1**).

**Figure 2:**
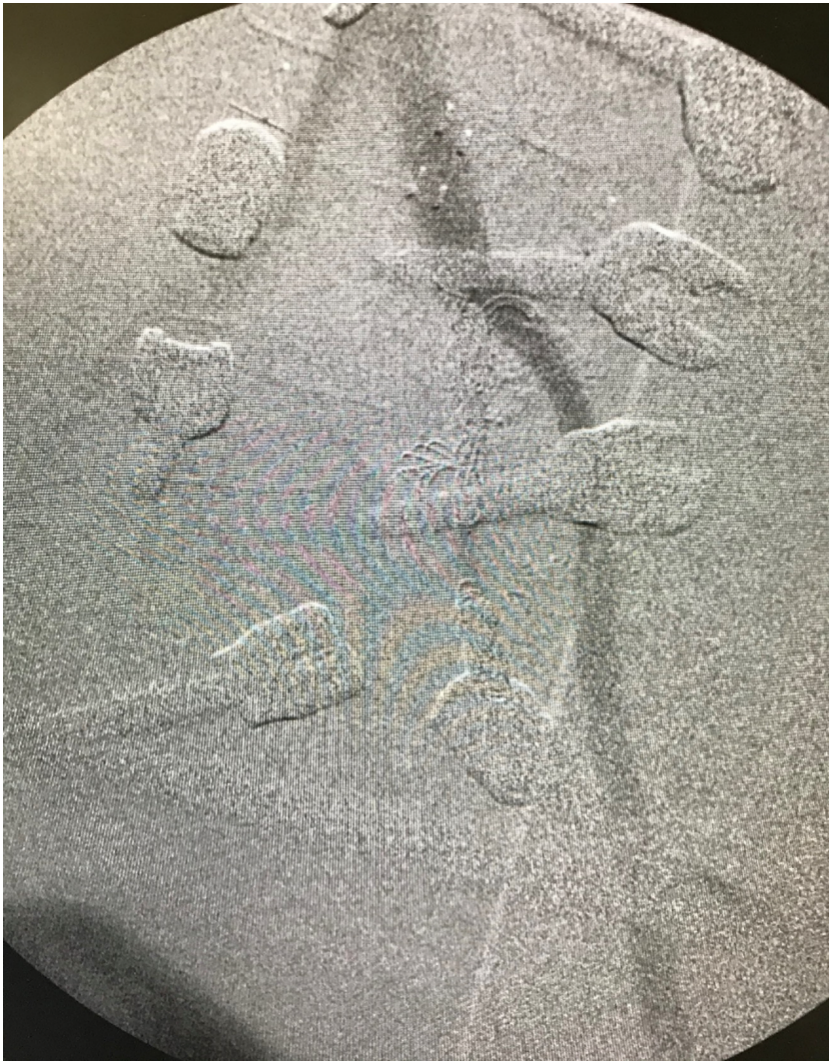
Fig. 2 The pseudoaneurysm disappeared after coil embolization using the isolation technique.

The right internal iliac artery was selected with a coaxial system consisting of a 4-Fr Cobra catheter (Terumo, Tokyo, Japan), a 2.2-Fr microcatheter (Coiling Support; Goldcrest Medic Co., Tokyo, Japan), and a microguidewire (AQUA V III; Johnson & Johnson, New Brunswick, NJ, USA). Coil embolization using the isolation technique was performed for the right internal iliac artery using a micro-coil (DELTAMAXX; Johnson & Johnson) (**Fig. 2**). In addition to noticing that the patient had abdominal distension, leading to suspicion of abdominal compartment syndrome, adequate ventilation volume could not be maintained and was complicated by acute respiratory failure (type II) based on blood gas levels; although her blood pressure improved after coil embolization of the right internal iliac artery.

While the bleeding was eventually stopped with compression of the puncture site for over 30 min in the prone and supine positions, the discectomy wound was immediately closed, and the patient was maintained in the supine position for laparotomy due to the abdominal compartment syndrome. After evacuating the large retroperitoneal hematoma, her vital signs resolved completely. The patient experienced multiple organ failure after the operation, so she was kept in the intensive care unit for seven days. She was discharged in good condition after rehabilitation.

## Discussion

Intraoperative injury of the great vessels during lumbar discectomy is extremely rare (0.014%–0.039%),^1)^ but is one of the most severe intraoperative complications and can be fatal if left untreated.

The factors affecting trauma mechanisms can be divided into surgical and anatomical factors.^2)^ Lumbar disc surgery is generally performed via a posterior approach in the prone position, where the intervertebral yellow ligament and degenerated intervertebral discs are cut and removed. Blind manipulation of the pituitary forceps during disk removal may cause them to slip through the anterior longitudinal ligament and reach the retroperitoneal space, resulting in vascular injuries.

Regarding anatomical factors, given the poor mobility of vessels, it is easy to damage the inferior vena cava and abdominal aorta at the L2/4 level and the iliac artery and vein at the L5 to S1 level.

In the present case, the right internal iliac artery was injured during disk removal at the L4 level. A similar case in which the internal iliac artery was injured during posterior lumbar disk surgery at the L4 to S1 level has been reported.^3)^ According to the literature, the risk factors for vascular injury during lumbar disk surgery include: 1) a history of abdominal radiotherapy; 2) peridiscal fibrosis; 3) recurrent disk surgery; 4) hernia-toward-abdomen disk; and 5) anatomical variations. The background of this patient’s lumbar disk and spine degeneration might have been a discrepancy compared with the normal anatomical position.^4,5)^

The clinical symptoms of vascular injuries can be divided into the acute and chronic phases.^6)^ Acute symptoms result from massive blood loss leading to hypotension and tachycardia. In addition, an excessively large retroperitoneal hematoma in the supine position can lead to abdominal compartment syndrome. The hemodynamics of the patient in the present case were unstable until evacuation of the hematoma was achieved after coil embolization of the right internal iliac artery. On the other hand, chronic symptoms may occur due to the formation of an arteriovenous fistula,^2)^ which can cause swelling of the legs, fatigue, and dyspnea on effort because of the formation of an arteriovenous shunt.

The treatment includes direct suture closure with laparotomy, grafting, or patch closure of the fistula.^7)^ Due to hemodynamic instability in the present case, we decided to perform endovascular therapy via a popliteal artery approach in the prone position because diagnosis and treatment can be carried out simultaneously, and it has a shorter procedure time than open repair, where the approach to the injured vessels would be more difficult.

Even if a stent graft can be used, distal embolization is required, which takes a substantial amount of time. Furthermore, no plugs are available in an emergency situation; however, glue may be a viable option if there are not enough coils to complete the embolization.

Although a transpopliteal approach in the prone position is typically used for peripheral arterial diseases, it can also be applied to more complicated situations, such as the present case. However, it should be kept in mind that the orifice of the internal iliac artery faces anteriorly in the supine position, which can assist the catheterization.

Having a good understanding of this endovascular option may provide surgeons with the best chance to treat this rare but critical condition during lumbar disk surgery.

## Conclusion

Life-threatening vascular injury can occur during lumbar disk surgery. A transpopliteal endovascular approach in the prone position may provide the best chance to treat this rare but critical condition.

## Additional Note

Written informed consent was obtained from the patient for publication of the case details.

## Additional Remarks

This case report was presented at the 59th Annual Meeting of Japanese College of Angiology, Hiroshima, Japan, October 25–27, 2018.
